# Improved adherence with once-daily versus twice-daily dosing of mometasone furoate administered via a dry powder inhaler: a randomized open-label study

**DOI:** 10.1186/1471-2466-10-1

**Published:** 2010-01-05

**Authors:** David Price, Anne Robertson, Kevin Bullen, Cynthia Rand, Rob Horne, Heribert Staudinger

**Affiliations:** 1 Centre of Academic Primary Care, University of Aberdeen, Foresterhill Health Centre, Aberdeen, UK; 2 Barrhead Health Centre, Barrhead, UK; 3 The Avenue Surgery, Wiltshire, UK; 4 Department of Medicine, Johns Hopkins University, Baltimore, MD, USA; 5 The School of Pharmacy, University of London, London, UK; 6 Schering-Plough Research Institute, Kenilworth, NJ, USA

## Abstract

**Background:**

Poor adherence with prescribed asthma medication is a major barrier to positive treatment outcomes. This study was designed to determine the effect of a once-daily administration of mometasone furoate administered via a dry powder inhaler (MF-DPI) on treatment adherence compared with a twice-daily administration.

**Methods:**

This was a 12-week open-label study designed to mimic an actual clinical setting in patients ≥12 years old with mild-to-moderate persistent asthma. Patients were randomized to receive MF-DPI 400 μg once-daily in the evening or MF-DPI 200 μg twice-daily. Adherence was assessed primarily using the number of actual administered doses reported from the device counter divided by the number of scheduled doses. Self-reports were also used to determine adherence. Health-related quality of life, healthcare resource utilization, and days missed from work or school were also reported.

**Results:**

1233 patients were randomized. The mean adherence rates, as measured by the automatic dose counter, were significantly better (*P *< 0.001) with MF-DPI 400 μg once-daily in the evening (93.3%) than with MF-DPI 200 μg twice-daily (89.5%). Mean adherence rates based on self-reports were also significantly better (*P *< 0.001) with MF-DPI 400 μg QD PM (97.2%) than with MF-DPI 200 μg twice-daily (95.3%). Adherence rates were lower in adolescents (12-17 years old). Health-related quality of life improved by 20% in patients using MF-DPI once-daily in the evening and by 14% in patients using MF-DPI twice-daily. Very few (<8%) patients missed work/school.

**Conclusion:**

Mean adherence rates were greater with a once-daily dosing regimen of MF-DPI than with a twice-daily dosing regimen.

This trial was completed prior to the ISMJE requirements for trial registration.

## Background

It is well established that inhaled corticosteroid (ICS) therapy is the most effective treatment for patients with persistent asthma[1,2]. Unfortunately, studies have shown adherence with prescribed ICS therapy is poor, with adherence rates generally thought to range from 30%-70% [3-6]. Low adherence is not only a major barrier to achieving positive treatment outcomes in asthma management, but may also be associated with an increased risk of death due to asthma[7] and increased health care costs, primarily through overuse of urgent care, hospitalization, and unnecessary physician visits[5,8,9].

Although further investigation on the effects of once-daily dosing on ICS treatment adherence under more controlled settings are needed, better adherence has been observed in asthma[10] and other disease treatments with once-daily dosing regimens than with twice-daily regimens [11-13]. A case-control study by Guest et al.[10] found that patients on twice-daily asthma treatments who were switched to once-daily treatments were better compliers than those switched to another twice-daily treatment. In addition, primary care management costs were lower in patients who showed high adherence after switching to once-daily treatment.

Mometasone furoate administered via a dry powder inhaler (MF-DPI) has been shown to be effective in treating asthma, as shown in comparative studies with other ICSs [14-18]. Although early studies of MF-DPI in mild and moderate asthma patients evaluated twice-daily dosing regimens,[14,19] more recent studies have demonstrated the comparability of MF-DPI 400 μg once-daily in the morning to MF-DPI 200 μg twice-daily[20,21] and the high efficacy of once-daily dosing in the evening [22-25]. However, it should be noted that clinical studies assessing adherence are often complicated by a variety of factors, such as greater adherence rates due to close patient monitoring, which results in adherence rates not typical of clinical practice[13,26].

The primary objective of this study was to determine the effect of once-daily versus twice-daily dosing of MF-DPI on treatment adherence in a study designed to mimic the actual clinical setting.

## Methods

### Patient Population

This multicenter study (143 sites in the United Kingdom) enrolled adolescents and adults aged 12 years or older with mild-to-moderate persistent asthma, and a diagnosis of asthma for at least 1 year. The study had institutional review board approval at all sites, and all patients provided informed consent before any study procedures were conducted. The first visit date was February 5, 2003, and the latest visit/contact date was February 26, 2004. Eligible patients had been treated with either beclomethasone dipropionate (BDP) hydrofluoroalkane (≤500 μg/d) or BDP chlorofluorocarbon (≤1000 μg/d) for ≥12 weeks, and had a stable BDP dosing regimen for ≥4 weeks immediately before study entry. The inclusion of patients who used BDP as their prior ICS therapy was justified because BDP was the ICS prescribed most commonly in the UK when the study was conducted, thereby providing a patient population as large and homogeneously-treated as possible. Eligible patients had no clinically significant disease that would interfere with study evaluations, and female patients of childbearing potential were required to use medically accepted birth control. Patients who required ventilator support for respiratory failure due to asthma within the previous 5 years or were hospitalized within the previous 3 months because of asthma were ineligible.

### Study Protocol

This was a 12-week, open-label study designed to mimic an actual clinical setting. Visits took place every 4 weeks. Patients were randomized to receive either MF-DPI 400 μg once-daily in the evening or MF DPI 200 μg twice-daily from inhalers measuring 220 μg/actuation and delivering 200 μg/inhalation. Assessments were made at baseline, and weeks 4, 8, and 12. Patients were instructed in inhaler use and peak flow measurement to demonstrate proficiency and received salbutamol for rescue medication. Optional diary cards were provided to record adverse events (AEs), use of rescue medication, and changes in concomitant medication between visits. Completed diary comment cards were reviewed at all subsequent visits. Following increases in asthma symptoms from baseline, patients used the peak flow meter to obtain an objective measure of asthma worsening. Daily use of the peak flow meter was not required to more closely mimic real-life practice. Patients were also instructed to follow an asthma action plan based on their personal best peak flow at the first visit. If peak flow was <75% personal best for 2 consecutive days, the patient was required to consult the investigator. Recommended clinical practice guidelines were also provided to investigators.

### Primary Evaluation

Adherence was calculated as the number of administered doses (as determined by device counter number) times 100 divided by the number of scheduled doses. Data were not included for analysis if invalid (eg, gross misuse of device, missing treatment end dates, or device malfunction). Examples of gross misuse of the device included failure to twist the cap when opening/closing the inhaler, or removing and replacing the inhaler cap more than once without taking a dose. If a device was not returned, it was assumed to have been unused.

### Secondary Evaluations

Secondary evaluations included measurements of adherence based on the patients' or their guardians' reports of adherence, regardless of the actual counter readings. The physician's assessment of a therapeutic response was documented at all visits. Health-related quality of life (HRQOL) was assessed using the Integrated Therapeutics Group Asthma Short Form (ITG-ASF), a brief and reliable disease-specific questionnaire[27]. For this study, the ITG-ASF was administered at baseline and week 12. The ITG-ASF does not have a derivation of total score for children, therefore, only HRQOL data obtained from subjects at least 16 years old are reported. Healthcare resource utilization and the number of days missed from work or school were recorded at all visits.

### Safety Evaluation

Safety assessments included reporting of AEs at all visits. An abbreviated physical exam and vital signs (heart, lungs, weight, blood pressure, pulse, and breath rates) were performed on visits 1 and 4. An evaluation of asthma worsening was performed at all visits, with asthma worsening being defined as an increased use of rescue medication (>12 inhalations on 2 consecutive days), a decrease in peak flow of >25% on 2 consecutive days, or clinical asthma exacerbations (unscheduled doctor's visit, hospitalization, ER visit, and/or use of additional asthma medications other than short-acting β-agonists).

### Statistical Analyses

A total of 1300 patients, or 650 per treatment group, were targeted for inclusion in this study, allowing a 90% power to detect a difference of at least 10% between distributions of adherence rates. The study was not powered to test differences in mean adherence. A sample of this size also allowed a sub-group analysis, with a 90% power to detect positive correlations of more than 0.15 between the level of adherence and the total ITG-ASF within each group (0.05 significance level). The Kolmogorov-Smirnov test was used to determine the significance of observed differences in the distribution of adherence rates between the 2 treatment groups. The Pearson correlation coefficient was used to analyze the correlation between adherence and changes in the ITG-ASF score. The chi-square test was used to analyze health care utilization and number of missed days of school/work. An analysis of variance (ANOVA) was used to extract sources of variation by treatment and center.

## Results

### Patients

**Figure 1:**
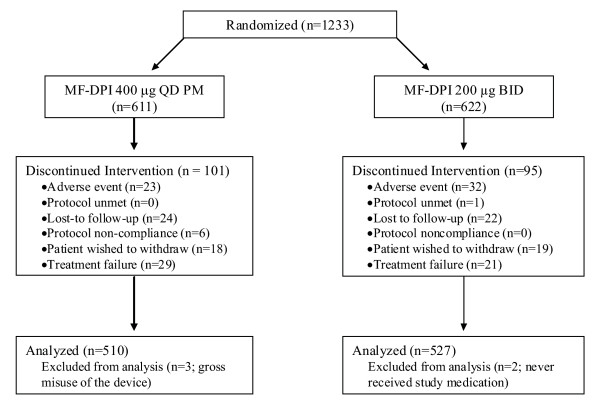
**Subject Disposition**. MF-DPI = mometasone furoate administered via a dry powder inhaler.

A total of 1233 patients were randomized to treatment; 611 patients in the MF-DPI 400 μg once-daily group and 622 patients in the MF-DPI 200 μg twice-daily group (Figure 1). The primary analysis of adherence excluded subjects with invalid data, subjects missing treatment end dates, and subjects who reported device issues. Adherence data based on the device counter was analyzed for 557 patients in the MF-DPI 400 μg once-daily group and 578 patients in the MF-DPI 200 μg twice-daily group. Similar proportions of subjects in the 2 treatment groups returned all 6 canisters of study medication: 92.0% (469/510) in the MF-DPI 400 μg once-daily group and 90.7% (478/527) in the MF-DPI 200 μg twice-daily group. No statistical differences were present between the randomized groups at baseline in age, sex, race, and weight, or in duration of disease (seasonal or allergic rhinitis or asthma). Baseline demographics are shown in Table 1.

**Table 1:** Baseline Demographics of Patients

	MF-DPI 400 μg QD PM (n = 611)	MF-DPI 200 μg BID (n = 622)
Mean age, y	50.9	50.2
		
Patients by age, n (%)		
12 to <18 y	21 (3)	22 (4)
18 to <65 y	444 (73)	465 (75)
≥65 y	146 (24)	135 (22)
		
Sex		
Women/men	338/273	357/265
		
Race		
White/nonwhite	607/4	615/7
		
Mean body weight, kg	77.3	76.7
	n = 599	n = 614
		
Mean duration of SAR, y	17.2	19.2
	n = 238	n = 235
		
Mean duration of PAR, y	19.7	20.9
	n = 163	n = 164
		
Mean duration of asthma, y	16.4	16.2
	n = 611	n = 622

BID = twice-daily; MF-DPI = mometasone furoate administered via a dry powder inhaler; PAR = perennial allergic rhinitis; QD PM = once-daily in the evening; SAR = seasonal allergic rhinitis.

### Primary Endpoint (Adherence)

**Figure 2:**
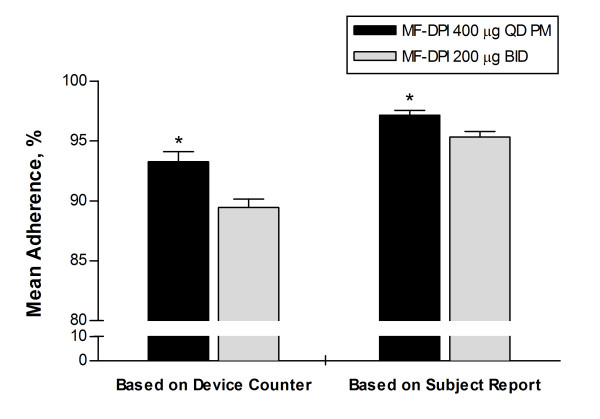
**Mean adherence to treatment**. Adherence was calculated as administered doses divided by scheduled doses × 100, as measured by dose counter and patient self-report. Error bars represent standard error of the mean. *P < 0.001. BID = twice-daily; MF-DPI = mometasone furoate administered via a dry powder inhaler; QD = once-daily.

The primary endpoint of adherence as measured by device counter was 93.3% (95% CI: 91.6-94.9) for the once-daily group and 89.5% (95% CI: 88.1-90.8) for the twice-daily group; the maximum difference in distribution of adherence rates results was significant at *P *< 0.001 (Figure 2). As measured by patient self-reports, adherence was 97.2% (95% CI: 96.4-97.9) for the once-daily group and 95.3% (95% CI: 94.4-96.2) for the twice-daily group, and the difference in distribution of the adherence results was significant at *P *< 0.001 (Figure 2). In the subgroup of patients who received and returned all 6 canisters of study medication, adherence was 96.4% (95% CI: 95.5-97.3) for the once-daily group (n = 469), and 91.8% (95% CI: 91.0-92.7) for the twice-daily group (n = 478; *P *< 0.001). Adherence was numerically lower for 12-18 year olds (once-daily group = 80.8% [95% CI: 64.1-97.5]), twice-daily group = 87.3% [95% CI: 81.2-92.4]); these patients comprised only 3% of the population, and statistical analysis was not done because there were very few patients in this subgroup. The rates of adherence for female and male patients were similar to each other and to the overall population.

### Secondary Endpoints

**Figure 3:**
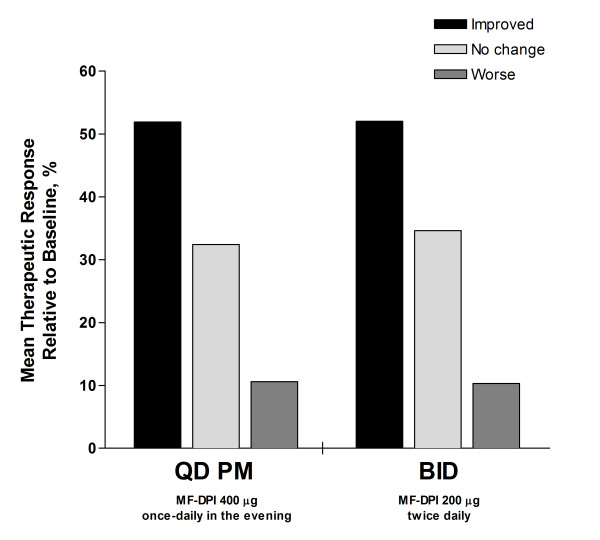
**Physician's evaluation of therapeutic response**. Response expressed as mean relative to baseline. *P *= not significant. MF-DPI = mometasone furoate administered via a dry powder inhaler.

**Figure 4:**
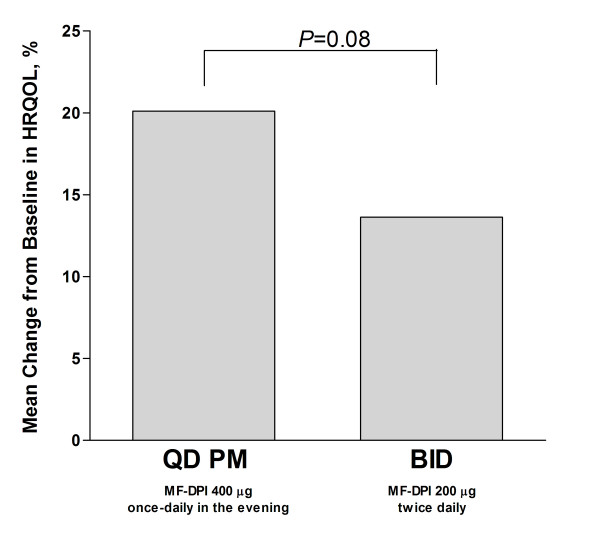
**Mean percent change in HRQOL**. Changes from baseline in HRQOL were measured by ITG-ASF scores in subjects ≥16 years of age. HRQOL = health-related quality of life; ITG-ASF = Integrated Therapeutics Group-Asthma Short Form; MF-DPI = mometasone furoate administered via a dry powder inhaler; QD = once-daily.

Using 2-way ANOVA with treatment and site effects, 52% of individual patients showed improvement in the physician's evaluation of therapeutic response on a scale of 1-5 from baseline, with 1 representing much improved and 5 representing much worse (*P *= not significant; Figure 3). With regard to HRQOL as measured by ITG-ASF scores (assessed in subjects at least 16 years of age), both groups improved relative to baseline (Figure 4). No significant correlation existed between HRQOL and adherence. However, there was a numerically greater increase in HRQOL in the once-daily group (20%) relative to the twice-daily group (14%), although the difference was not statistically significant (*P *= 0.08). Regarding healthcare utilization, few patients had missed days of work or school (once-daily group = 9; twice-daily group = 10) or unscheduled health visits (once-daily group = 37; twice-daily group = 33). No significant difference was seen between treatment groups (*P *≥ 0.48).

### Safety Evaluation

The nature and occurrence of AEs were similar to those reported in other studies of ICS [28-30]. AEs for the most part were not considered treatment related, and were mild-to-moderate in severity (Table 2). Only 6 patients reported severe treatment-related AEs; 1 patient each with mouth ulceration and oral candidiasis in the MF-DPI 400 μg once-daily group, and 1 patient each with headache, tongue disorder, oral candidiasis, and hypoglycemia in the MF-DPI 200 μg twice-daily group. Fifty-five patients (23 in the once-daily group and 32 in the twice-daily group) discontinued treatment owing to AEs, and were not analyzed. An additional 38 patients had treatment interrupted owing to AEs. Two patient deaths were reported (1 due to cardiorespiratory arrest and 1 due to lung carcinoma); neither was considered treatment related.

**Table 2:** Incidence of Treatment-Related AEs Reported by ≥1% of Patients

	MF-DPI 400 μg QD PM (n = 611)	MF-DPI 200 μg BID (n = 622)
Patients reporting any AE, n (%)	85 (14)	109 (18)
Headache	10 (2)	12 (2)
Dysphonia	3 (<1)	9 (1)
Mouth dry	12 (2)	11 (2)
Mouth ulceration	5 (1)	1 (<1)
Candidiasis, oral	9 (1)	16(3)
Pharyngitis	16 (3)	15 (2)
Upper respiratory tract infection	5 (1)	0
Cough	2 (<1)	6 (1)
Hoarseness	4 (1)	5 (1)
Throat dry	11 (2)	3 (<1)
Rash	0	4 (1)

AE = adverse event; BID = twice-daily; MF-DPI = mometasone furoate administered via a dry powder inhaler; QD PM = once-daily in the evening.

## Discussion

Mean adherence rates, as measured by the automatic dose counter, were found to be significantly better for a once-daily regimen of MF-DPI compared to twice-daily administration. Once-daily treatment with MF-DPI also significantly improved mean adherence rates based on patient self-reports compared with twice-daily MF-DPI, as well as mean adherence rates based only on those patients who received and returned all 6 canisters of study medication.

Despite the significant differences in mean adherence, we found no significant differences in clinical outcomes. This is likely due to the unusually high rates of adherence observed in both study groups (89%-93%). The adherence rates in this study are much higher than the rates previously reported in the literature (30% to 70%),[5,6] and may have been influenced by the constraints of the clinical trial in terms of attention and study participants. Direct interaction between physicians and asthma patients (or their parents/caregivers) has been shown to improve adherence. In one study, a treatment group receiving direct feedback from clinicians had an adherence rate of approximately 60% at the first visit, which then increased and remained above 70% at each weekly visit for the duration of the 10-week study. In the control group receiving usual asthma care, adherence declined from approximately 50% at the first weekly visit to <30% at the final weekly visit[4]. In addition, patients who volunteer to participate in a study may be more willing to comply with a treatment regimen.

This study was designed to prevent some of these issues. Patients were not informed that device dose counts were checked at each visit. The study also tried to decrease aspects of a clinical study that would promote adherence and tried to mimic asthma management in a typical clinical practice. Doctor visits were limited to once a month, and patients were not required to keep daily records of symptoms or peak flow. In addition, in accordance with clinical practice guidelines,[1,2] patients were given an "Asthma Action Plan" to help guide them in self-managing their symptoms.

A limitation of this study may be its duration. Treatment adherence in clinical trial settings does not typically reflect normal rates of adherence until a more prolonged period has passed[31]. One long-term study of children and adolescents (ages 7-16 y) with mild asthma treated with once- or twice-daily doses of ICS found that adherence rates significantly declined following 3 months of treatment and continued to decline by more than 50% in both treatment groups after 9 months[31]. Thus, it is possible that given a longer duration, the adherence rates observed in this study would decrease to lower levels. Furthermore, this study did not use direct measures of adherence, which are not practical or reliable for large numbers of patients in ordinary settings. It is not possible to document whether patients were actually taking their medication correctly or following their dosing regimen. The patients may have taken multiple doses at the same time, or patients may have actuated their inhaler but not taken the medication. In addition, since patients were not required to keep daily diary records, their self-reports were based largely on memory, which may have resulted in erroneous adherence estimates.

The literature is lacking clear documentation on the impact of once- vs twice-daily dosing. However, reliable measurements of medication use were obtained in a study that evaluated adherence by patients with mild asthma randomized to treatment with oral montelukast 10 mg once-daily (n = 189) or inhaled fluticasone propionate 88 μg twice-daily (n = 191)[32]. The montelukast pill boxes and fluticasone inhalers used in the study had electronic monitoring devices to record medication use continuously over 12 weeks of placebo-controlled double-blind treatment and 36 weeks of open-label treatment with no placebo. Adherence rates with once-daily montelukast and twice-daily fluticasone were 77.5% and 70.2%, respectively (*P *< 0.0001), during double-blind treatment and 71.4% and 63.9%, respectively (*P *= 0.001), during open-label treatment. Although adherence rates in the study were less than prescribed, it was found that adherence with once-daily treatment was superior to adherence with twice-daily treatment. This difference could be related to the fact that montelukast is an oral medication and fluticasone is an inhaled medication.

Despite limitations of the present study, the observed efficacy results suggest that patients were taking their medication and that their asthma was more adequately treated than it had been prior to randomization. Both treatment groups showed improved HRQOL, and over half of all patients in both treatment groups were rated as having an improved therapeutic response from baseline. Very few patients had unscheduled office or home visits or reported missed days from work or school. The differences in adherence between the once-daily and twice-daily groups were statistically significant, yet they were small and not correlated with improvements in HRQOL. Moreover, once-daily dosing in adolescents was associated with numerically lower adherence than twice-daily dosing, although this difference was not statistically significant.

Asthma therapy is complex, and treatment dosing frequency is one of many factors that affect adherence. Both groups in the present study had high rates of adherence, which suggests that factors including the physician-patient relationship, medical costs, and patient beliefs regarding illness and medication strongly influence adherence in clinical practice[33,34]. According to the model proposed by Horne et al,[33] treatment adherence is related to a patient's perception of illness. Strong correlations have been observed between (1) nonadherence and patients who believed treatment medication was unnecessary or had concerns of adverse effects and (2) greater adherence and patients who believed their asthma was of greater severity[33]. It is essential to individualize therapy to meet patient needs by identifying nonadherence and the causes of nonadherence for individual patients, as recommended in a recent paper by the International Primary Care Respiratory Group.[35]

One indication for prescribing once-daily asthma therapy is for patients who are likely to experience difficulties with twice-daily therapy. It is clear that most patients randomized to twice-daily treatment in the present study did not find this regimen to be a barrier to adherence, although overall adherence rates in clinical practice appear to be significantly lower than adherence rates in this trial. Further studies are needed to determine the degree to which twice-daily dosing might erode adherence over time, and to measure the effects of switching to a once-daily regimen for those patients who find that twice-daily therapy is a barrier to adherence. Although for some patients it is also possible that dosing frequency may have an impact on treatment efficacy, given that a once-daily medication has only one trough per day, whereas a twice-daily medication has two troughs per day. In the present study, however, the once- and twice-daily doses of MF-DPI had similar efficacy.

## Conclusion

Overall adherence to treatment with mometasone furoate dry powder inhaler was very high in this study, with a statistically greater mean adherence rate using a once-daily dosing regimen versus a twice-daily dosing regimen.

## Abbreviations

AE: adverse event; ANOVA: analysis of variance; BDP: beclomethasone dipropionate; BID: twice daily; HRQOL: health-related quality of life; ICS: inhaled corticosteroid; ITG-ASF: Integrated Therapeutics Group Asthma Short Form; MF-DPI: mometasone furoate administered via a dry powder inhaler; QD PM: once-daily in the evening.

## Competing interests

Dr. Price or his team have received grants and research support from UK National Health Service, Altana Pharma, AstraZeneca, Boehringer Ingelheim, GlaxoSmithKline, Merck, Sharpe and Dohme, Novartis, Pfizer, Schering Corp., a division of Merck & Co., and Teva; has consultant arrangements with Boehringer Ingelheim, GlaxoSmithKline, Merck generics, Merck, Sharpe and Dohme, Novartis, Schering Corp., a division of Merck & Co., and Teva; and has spoken for Altana Pharma, Boehringer Ingelheim, GlaxoSmithKline, Merck, Sharpe and Dohme, Pfizer, and Schering Corp., a division of Merck & Co., Dr. Robertson declares no competing interests. Dr. Bullen declares no competing interests. Dr. Rand has served on the Schering-Plough Respiratory Leadership Council and on the Merck Childhood Asthma Network advisory board. Dr. Horne has received grants from Gilead Life Sciences and Hayward Medical Communications/Shire Pharmaceuticals, and has consulted for various pharmaceutical companies. He is an officer of the University of London, School of Pharmacy. Dr. Staudinger is an employee and has stock and stock options for Schering Corp., a division of Merck & Co.

## Authors' contributions

Drs. DP, AR, KB, CR, and RH contributed to the acquisition, analysis, and interpretation of the data. Drs. DP, CR, and RH contributed to the design of the study. Dr. HS contributed to the design of the study and interpretation of the results. All authors critically revised the manuscript for intellectual content and approved the final version.

## Pre-publication history

The pre-publication history for this paper can be accessed here:

http://www.biomedcentral.com/1471-2466/10/1/prepub
